# Parental determinants associated with early growth after the first year of life by race and ethnicity

**DOI:** 10.3389/fped.2023.1213534

**Published:** 2023-07-25

**Authors:** Liliana Aguayo, Cecilia Chang, Luke R. McCormack, Madeleine U. Shalowitz

**Affiliations:** ^1^ Hubert School of Global Health, Emory University Rollins School of Public Health, Atlanta, GA, United States; ^2^ Research Institute, NorthShore University HealthSystem, Evanston, IL, United States; ^3^ Rush Medical College of Rush University Medical Center, Chicago, IL, United States; ^4^ Department of Psychiatry & Behavioral Sciences, Rush University Medical Center, Chicago, IL, United States

**Keywords:** infant, obesity, childhood obesity, father, parental education, Hispanic/Latinx

## Abstract

**Objective:**

To investigate maternal and parental factors associated with changes in children's body mass index percentile (BMI-P) from 12 to 24 months.

**Methods:**

Data from a prospective cohort of racially and ethnically diverse mothers, fathers, and children (*n* = 245) were used. Changes in BMI-P from 12 to 24 months of age were examined using height and weight measurements collected at both times. Separate longitudinal mixed-effects models with maximum likelihood were introduced to examine the determinants introduced by mothers and determinants from both parents among all children, and by race and ethnicity.

**Results:**

Models that examine maternal and parental factors showed that children's overall BMI-P decreased from 12 to 24 months [*β *= −4.85, 95% confidence interval (CI), −7.47 to −2.23]. Stratified tests showed that White children whose parents graduated high school or completed a 4-year college degree or higher had greater decreases in BMI-P than White children born to parents with less than high school education (*β *= −60.39, 95% CI, −115.05 to −5.72; *β *= −61.49, 95% CI, −122.44 to −0.53). Among Hispanic/Latinx children, mean BMI-P significantly decreased from 12 to 24 months (*β *= −7.12, 95% CI, −11.59 to −2.64). Mother's older age (*β *= 1.83, 95% CI, 0.29–3.36) and child female sex (*β *= 11.21, 95% CI, 1.61–20.82) were associated with gains in children's BMI-P, while father's older age was associated with decreases (*β *= −1.19, 95% CI, −2.30 to −0.08).

**Conclusions:**

Parental determinants associated with children's early growth varied by children's sex and racial and ethnic background. Results highlight the importance of understanding racial and ethnicity-specific obesity risks and including fathers in research.

## Introduction

In the United States, by the time Hispanic/Latinx and non-Hispanic Black children enter kindergarten, they already bear a higher prevalence of elevated body mass index percentile (BMI-P) than White children (1). Excessive weight gain during the first years of life often continues into adulthood triggering the development of racial and ethnic disparities in cardiometabolic diseases (2). Children who experienced rapid weight gain—particularly during the first 24 months of life—are more likely to develop obesity and adverse cardiometabolic health by early adolescence, than children who never exceeded the 85th BMI-P in the first 2 years of life (3, 4).

Maternal traits, such as mother's BMI, and social determinants of health, such as education and socioeconomic status, are strong predictors of childhood obesity (5–7). Beyond these maternal determinants, studies examining other familial factors yielded inconsistent findings (7–9). The inconsistency in findings may stem from differences across racial and ethnic groups in both child growth patterns and the contribution of social determinants to excessive weight gain in early life (7). Previous research has shown that higher income and education is associated with a lower BMI among White but not Black or Mexican American adults (10). Similarly, Black and Hispanic/Latinx youth experienced diminishing returns in health with increasing parental socioeconomic status relative to White youth (10–13). These differences may begin early in life. Several studies conducted with the Early Childhood Longitudinal Study Birth Cohort (ECLS-B) dataset, a nationally representative cohort of US children, showed that growth trajectories of US 4–6-year-old children vary by race and ethnic background and language spoken at home (14). Among Hispanic/Latina girls, weight differences were mostly explained by the difference in socioeconomic status when compared with their White counterparts (14). Rapid weight gain during the first 9 months of life was the biggest predictor of obesity at kindergarten entrance for White and Black children, but not for Hispanic/Latinx children (15). Notably, the characterization of household socioeconomic status in the ECLS-B study accounted for father's educational attainment, occupation, and prestige. Undoubtedly, although fathers exert an important influence in children's lives and obesity risks, the influence of fathers’ social determinants on children's health is much less understood (16). There are data suggesting that maternal–paternal combined traits would allow improved prediction of early childhood obesity (17), but research of paternal determinants remains limited (16).

These research gaps are a critical barrier to the development of effective obesity prevention strategies for racially and ethnically diverse children, particularly in the first 2 years of life. During the period from 12 to 24 months of age, children with healthy growth trajectories display a decrease in adiposity after they begin to walk and expend more energy (18, 19). In contrast, children who gain excessive weight during this period are at increased risks for delayed motor development (18), future obesity (2, 20), elevated cardiometabolic risk (3, 4), and associated premature diseases and morbidity in older ages (21). While rapid weight gain is considered a critical risk factor during the first 2 years of life, research suggest that weight gain after the first 12 months of life could have more profound effects than weight gains in earlier periods of infancy. In a previous study, researchers found that weight gain during the first 4 months of life was not associated with future obesity risks (22). In contrast, weight gain from birth to 12 months was associated with higher likelihood of obesity by 3 years of age (22). The authors did not examine the impact of weight gain after 12 months. Further information is needed to better understand children's BMI-P dynamics during the period from 12 to 24 months of age and identify differences in the risk factors associated with excessive weight gain among racial and ethnic minority children (23).

The purpose of this study is to investigate maternal and paternal determinants associated with changes in BMI-P from 12 to 24 months. We hypothesize that race- and ethnicity-stratified models will reveal differences in the benefits conferred by parental socioeconomic resources, and that when compared with models examining maternal determinants only, assessment of maternal–paternal combined determinants will introduce additional factors associated with changes in child's BMI-P from 12 to 24 months of age. In addition, we expect maternal and paternal BMI changes (when their child transitions from infancy to toddler ages) will be associated with their child changes in BMI-P from 12 to 24 months of age.

## Methods

### Study design and participants

Data from the Community Child Health Research Network (CCHN), a 5-year, multi-site prospective cohort study, were used. The CCHN study recruited African American/Black, Hispanic/Latinx, and White mothers and fathers of newborns at the time of delivery and followed them over 2 years. Participants were recruited in Washington, DC; Baltimore, MD; and Lake County, IL. Interviews were conducted when children were about 6, 12, and 24 months of age. Our analytical sample included data from the families that included three participants (mother, father, and child), with complete demographic data collected at baseline, and child BMI-P measurements collected at both the 12- and 24-month follow-up visits (*n* = 245). Fathers not cohabitating with mothers who agreed to participate in the study were included. Families with children born preterm, mothers not living with or actively in contact with the child delivered at the time of recruitment, and fathers reported as unknown, unavailable (e.g., incarcerated), or unwilling to participate in the study were excluded from the analytical sample.

Ethics approval was sought from and given by the Institutional Review Boards at the respective institutions affiliated with each of the study sites. Mother and father participants provided a written informed consent, which included permission to collect and access health information of themselves and their child. Additional details of the study have been published elsewhere (24).

### Measures

#### Outcome

##### Children's BMI-P measured at 12 and 24 months

Children's height and weight were measured when children were about 12 months old and a second time at about 24 months of age. Measurements were collected by trained field interviewers who followed a standardized protocol. Children's BMI-P was calculated based on World Health Organization (WHO) age- and sex-specific growth charts. Data of children who did not participate in the 12-month follow-up (*n* = 14) were imputed using measurements from the 6-month follow-up for estimations. Sensitivity analyses excluding these children are included in the Supplementary Materials. None of the children in our analytical sample were missing the 24-month height and weight measurements.

#### Independent variables

##### Parent's race, ethnicity, and nativity

Mothers and fathers were asked to self-identify their race and ethnicity using the following categories: African American or Black, White or Caucasian, and Hispanic or Latinx. Parents who did not report a single race were considered multiracial and combined with the “Other race” category. For nativity, mothers and fathers were asked to report if they were US or foreign-born. Responses were used to classify nativity using a dichotomous indicator for US vs. foreign-born.

##### Parent's BMI from children's 12–24 months of age

Trained interviewers measured mother's and father's height and weight when their child was 12 and 24 months old following a standardized protocol that included light clothing and no shoes. Height and weight measurements were used to compute BMI-P for sex and age. A BMI-P of 85th to under 95th percentile was considered overweight and 95th percentile and higher as obesity. BMI-P measurements were examined using continuous time-varying variables.

##### Cohabitation or marital status

Mothers were asked their cohabitation status and marital status with the child's father during first year of study. The responses of cohabitation or marital status were combined into two categories for “not married or living together” and “married or living together.”

##### Mother and father's level of education

Mothers and fathers were asked about their maximum level of education completed. Responses were combined in four categories for (1) less than high school; (2) high school, GED certificate, or technical school; (3) some college but not a 4-year degree; and (4) 4-year degree or higher, with less than high school as a reference. In the models that examined maternal and paternal predictors together, the highest level of education of both parents was used to examine the influence of parental education.

##### Family's poverty level

Mothers and fathers were asked to report their income. Individual's income was used to calculate family's income and corresponding poverty level. Poverty level was calculated based on percentage of Federal Poverty Level (FPL) for the aggregated family income and household size. Measurements were classified into three categories for ≤100% FPL, >100% to 200% FPL, and >200% FPL.

##### Covariates

Maternal and paternal age and children's demographic characteristics, specifically child's age, sex, and birth weight, were included as covariates. Data on these covariates were computed based on data obtained during the baseline interview.

### Statistical analysis

Baseline characteristics were examined overall and compared by children's race and ethnicity (White, Black, Latinx, and Other). Children's race was categorized based on the combined mother and father's race and ethnicity. Baseline categorical variables are displayed as frequency counts with percentages and were compared using chi-squared test or Fisher's exact test. Continuous variables are shown as means with standard deviations and were compared using ANOVA tests. Significant changes in continuous BMI-P of children from 12 to 24 months of age were examined using a set of longitudinal mixed-effects models with maximum likelihood estimating method and unstructured covariance. Data were fitted with random effects of intercept and time, which allowed us to account for multiple sources of variability in BMI-P changes from 12 to 24 months of age. Multivariable models examined the association of maternal predictors and predictors from both parents with changes in BMI-P from 12 to 24 months of age. Separate models were introduced to identify the contribution of mothers and the contribution from both parents. Tests included all children with complete families in our sample first, and in secondary analyses, we estimated models stratified by race and ethnicity. Given the sample size limitations, stratified tests were only conducted among White and Hispanic/Latinx children. Sensitivity analyses were conducted with a subsample (*n* = 231) that excluded 14 children for whom BMI-P was imputed. Characteristics of the subsample are introduced in Supplementary Table S1. *Post-hoc* power estimates revealed the analyses conducted with a sample of 231 families had a power of 77% to detect a difference of 0.15 in BMI-P while using an F-test with a significance level of 0.05 and adjusting for nine covariates. All statistical analyses were conducted in SAS 9.4 (SAS Institute, Cary, NC, United States).

## Results

### Characteristics of the study sample

Table 1 provides descriptive characteristics of the sample overall and by race and ethnicity. At 12 months, the mean (standard deviation) BMI-P of children was 61.3 (30.36), and 27.4% had a BMI-P at or above the 85th percentile. By 24 months, the mean BMI-P of children was 53.1 (32.3), and about 24.1% had a BMI-P at or above the 85th percentile. There were no significant differences in children's sex, birthweight, and weight status at 12 and at 24 months across racial and ethnic groups. Mothers’ and fathers’ mean age, nativity, level of completed education, poverty level, and marital status differ significantly by race and ethnicity.

**Table 1:** Child, mother, and father demographic characteristics, overall and by race and ethnicity.

	Overall	Non-Hispanic White	Non-Hispanic Black	Hispanic/Latinx	Other	*p* *
Child characteristics	*n* (%)/mean ± SD					
Child sex	245	80	23	104	38	
Male	127 (51.84)	40 (50.00%)	13 (56.52%)	55 (52.88%)	19 (50.00%)	0.939
Female	118 (48.16)	40 (50.00%)	10 (43.48%)	49 (47.12%)	19 (50.00%)	
Child birthweight						
Low	30 (12.24)	9 (11.25%)	4 (17.39%)	11 (10.58%)	6 (15.79%)	0.560
Normal	189 (77.14)	61 (76.25%)	16 (69.57%)	81 (77.88%)	31 (81.58%)	
Macrosomia	26 (10.61)	10 (12.50%)	3 (13.04%)	12 (11.54%)	1 (2.63%)	
Child weight status						
Child BMI-P at 12 months	61.3 (30.36)	60.85 ± 29.22	49.73 ± 35.80	62.42 ± 31.63	65.85 ± 24.84	0.388
Overweight or obesity at 12 months (BMI-P > 85th)	67 (27.35)	23 (28.75%)	3 (13.04%)	34 (32.69%)	7 (18.42%)	0.438
Child BMI-P at 24 months	53.14 (32.27)	52.63 ± 34.55	37.70 ± 29.61	56.10 ± 31.29	53.57 ± 29.79	0.527
Overweight or obesity at 24 months (BMI-P > 85th)	59 (24.08)	19 (23.75%)	3 (13.04%)	26 (25.00%)	11 (28.95%)	0.921
Parental characteristics						
Mother's age	(27.20 ± 5.67)	30.75 ± 4.73	25.91 ± 5.75	25.08 ± 4.71	26.28 ± 6.38	<0.001
Father's age	(29.73 ± 6.97)	32.44 ± 6.34	28.60 ± 6.33	27.99 ± 6.74	29.46 ± 7.66	<0.001
Mother's education						
Less than HS	53 (21.63)	2 (2.50%)	4 (17.39%)	42 (40.38%)	5 (13.16%)	<0.001
HS or equivalent	83 (33.88)	10 (12.50%)	9 (39.13%)	46 (44.23%)	18 (47.37%)	
Some college but not 4-year degree	45 (18.37)	17 (21.25%)	7 (30.43%)	11 (10.58%)	10 (26.32%)	
4-year degree or higher	58 (23.67)	48 (60.00%)	3 (13.04%)	3 (2.88%)	4 (10.53%)	
Other	6 (2.45)	3 (3.75%)	0 (0.00%)	2 (1.92%)	1 (2.63%)	
Father's education						
Less than HS	61 (24.9)	5 (6.25%)	5 (21.74%)	46 (44.23%)	5 (13.16%)	<0.001
HS or equivalent	95 (38.78)	19 (23.75%)	10 (43.48%)	48 (46.15%)	18 (47.37%)	
Some college but not 4-year degree	28 (11.43)	6 (7.50%)	6 (26.09%)	7 (6.73%)	9 (23.68%)	
4-year degree or higher	61 (24.90)	50 (62.50%)	2 (8.70%)	3 (2.88%)	6 (15.79%)	
Mother's poverty group						
≤100% FPL	68 (27.76)	4 (5.00%)	11 (47.83%)	39 (37.50%)	14 (36.84%)	<0.001
>100%–200% FPL	81 (33.06)	14 (17.50%)	5 (21.74%)	51 (49.04%)	11 (28.95%)	
>200% FPL	96 (39.18)	62 (77.50%)	7 (30.43%)	14 (13.46%)	13 (34.21%)	
Father's poverty group						
≤100% FPL	64 (26.12)	4 (5.00%)	7 (30.43%)	42 (40.38%)	11 (28.95%)	<0.001
>100%–200% FPL	57 (23.27)	14 (17.50%)	4 (17.39%)	33 (31.73%)	6 (15.79%)	
>200% FPL	124 (50.61)	62 (77.50%)	12 (52.17%)	29 (27.88%)	21 (55.26%)	
Marital or cohabitation status						
Not married or living together	27 (11.02)	3 (3.75%)	10 (43.48%)	9 (8.65%)	5 (13.16%)	<0.001
Married or living together	218 (88.98)	77 (96.25%)	13 (56.52%)	95 (91.35%)	33 (86.84%)	
Parental characteristics	*n* (%)/mean ± SD					
Mother's race and ethnicity						
Non-Hispanic White	89 (36.33)	80 (100.00%)	0 (0.00%)	0 (0.00%)	9 (23.68%)	<0.001
Non-Hispanic Black	26 (10.61)	0 (0.00%)	23 (100.00%)	0 (0.00%)	3 (7.89%)	
Hispanic/Latinx	123 (50.20)	0 (0.00%)	0 (0.00%)	104 (100.00%)	19 (50.00%)	
Other	7 (2.86)	0 (0.00%)	0 (0.00%)	0 (0.00%)	7 (18.42%)	
Father's race and ethnicity						
Non-Hispanic White	93 (37.96)	80 (100.00%)	0 (0.00%)	0 (0.00%)	13 (34.21%)	<0.001
Non-Hispanic Black	30 (12.24)	0 (0.00%)	23 (100.00%)	0 (0.00%)	7 (18.42%)	
Hispanic/Latinx	110 (44.90)	0 (0.00%)	0 (0.00%)	104 (100.00%)	6 (15.79%)	
Other	12 (4.90)	0 (0.00%)	0 (0.00%)	0 (0.00%)	12 (31.58%)	
Mother's nativity						
US born	149 (60.82)	74 (92.50%)	22 (95.65%)	23 (22.12%)	30 (78.95%)	<0.001
Foreign born	96 (39.18)	6 (7.50%)	1 (4.35%)	81 (77.88%)	8 (21.05%)	
Father's nativity						
US born	152 (62.04)	76 (95.00%)	21 (91.30%)	23 (22.12%)	32 (84.21%)	<.001
Foreign born	93 (37.96)	4 (5.00%)	2 (8.70%)	81 (77.88%)	6 (15.79%)	
Mother's weight status						
BMI (child's age 12 months)	28.25 (6.21)	27.79 ± 6.61	30.74 ± 8.31	28.20 ± 5.08	28.08 ± 7.10	0.396
Overweight or Obesity at 12 months (BMI > 25 kg/m^2^)	169 (69)	47 (58.75%)	18 (78.26%)	78 (75.00%)	26 (68.42%)	0.329
BMI (child's age 24 months)	28.2 (6.14)	27.58 ± 5.89	25.96 ± 5.51	29.00 ± 6.25	28.77 ± 6.72	0.461
Overweight or obesity at 24 months (BMI > 25 kg/m^2^)	165 (67.35)	49 (61.25%)	14 (60.87%)	75 (72.12%)	27 (71.05%)	0.548
Father's weight status						
BMI (child's age 12 months)	28.71 (4.83)	28.88 ± 5.00	29.76 ± 5.99	28.46 ± 4.48	28.45 ± 4.88	0.767
Overweight or obesity at 12 months (BMI > 25 kg/m^2^)	180 (73.47)	59 (73.75%)	16 (69.57%)	85 (81.73%)	20 (52.63%)	—
BMI (child's age 24 months)	28.86 (5.12)	28.76 ± 4.68	27.80 ± 6.17	29.11 ± 5.26	28.93 ± 5.76	0.925
Overweight or obesity at 24 months (BMI > 25 kg/m^2^)	185 (75.51)	61 (76.25%)	17 (73.91%)	80 (76.92%)	27 (71.05%)	0.308

HS, high school.

* *p*-value of comparisons across racial and ethnic groups carried out using chi-square/Fisher's exact tests (for categorical variables) and ANOVA (for continuous variables).

### Parental factors associated with change in children's BMI-P from 12 to 24 months

Table 2 presents the association of changes in children's BMI-P from 12 to 24 months with maternal factors separately and maternal and paternal factors combined. Models that examined maternal factors exclusively showed that when compared to being born to White mothers, children born to Hispanic/Latinx mothers had a significant increase in mean BMI-P from 12 to 24 months [*β *= 11.86, 95% confidence interval (CI), 0.75–22.97, *p *= 0.04]. Changes in children's BMI-P were associated with children's birthweight (*β *= 2.09, 95% CI, 0.00–4.18, *p *< 0.05), but not with changes in their mother's mean BMI over the same period of time (*β *= 0.36, 95% CI, −0.13 to 0.86, *p *= 0.15). Sensitivity analyses presented in Supplementary Table S2 showed that both associations were attenuated when examined in a subsample (*n* = 231) that excluded children for whom BMI-P was imputed in the 12-month visit.

**Table 2:** Association of changes in children's age- and sex-adjusted body mass index percentile (BMI-P) from 12 to 24 months with maternal factors and parental factors.

	Maternal factors			Parental factors^a^		
	Estimate	95% CI	*p* *	Estimate	95% CI	*p* *
Child factors	(*N* = 245)			(*N* = 245)		
Child BMI-P from 12 to 24 months	−5.08	−7.73 to −2.43	<0.001	−4.85	−7.47 to −2.23	<0.001
Child sex						
Male ref	—	—	—	—	—	—
Female	1.87	−4.68 to 8.42	0.572	2.72	−3.74 to 9.18	0.405
Birthweight	2.09	0.00 to 4.18	<0.05	2.03	−0.10 to 4.16	0.061
Parental factors						
Race and ethnicity						
Non-Hispanic White (ref)	—	—	—	—	—	—
Non-Hispanic Black	−7.36	−20.15 to 5.44	0.256	−5.44	−19.14 to 8.26	0.432
Hispanic/Latinx	11.86	0.75 to 22.97	0.037	5.00	−8.04 to 18.05	0.448
Other	−11.38	−31.78 to 9.02	0.271	6.42	−5.07 to 17.90	0.270
Nativity						
US born (ref)	—	—	—	—	—	—
Foreign born	−3.75	−13.05 to 5.55	0.425	5.10	−6.28 to 16.48	0.376
Other				−0.57	−12.58 to 11.44	0.925
Education (highest of 2)						
Less than HS	—	—	—	—	—	—
HS or equivalent	2.70	−6.73 to 12.12	0.571	0.83	−9.69 to 11.35	0.876
Some college but not 4-year degree	1.02	−11.72 to 13.75	0.874	1.81	−11.28 to 14.91	0.784
4-year degree or higher	−2.75	−18.12 to 12.62	0.723	2.25	−12.52 to 17.03	0.763
Poverty group (highest)						
≤100% FPL (ref)	—		—	—	—	—
>100%–200% FPL	−2.71	−11.51 to 6.10	0.543	−1.66	−11.83 to 8.50	0.746
>200% FPL	3.11	−8.99 to 15.20	0.611	−2.39	−12.89 to 8.11	0.653
Cohabitation status						
Never married or living together ref.	—		—	—		—
Married or living together	7.71	−4.24 to 19.66	0.203	7.10	−4.81 to 19.02	0.240
BMI time-varying						
Mother's BMI from 12 to 24 months	0.36	−0.13 to 0.86	0.148	0.36	−0.13 to 0.86	0.150
Father's BMI from 12 to 24 months				0.01	−0.63 to 0.64	0.980
Age at enrollment						
Mother	0.21	−0.52 to 0.94	0.571	0.63	−0.44 to 1.69	0.245
Father				−0.58	−1.39 to 0.22	0.155

ref., reference; HS, high school.

^a^ Parental factors included demographic characteristics of mother and father.

* *p*-value of the longitudinal mixed-effects models with maximum likelihood estimating method and unstructured covariance.

Models that examined mothers’ and fathers’ predictors combined confirmed that children's overall mean BMI-P significantly decreased from 12 to 24 months (*β *= −4.85, 95% CI, −7.47 to −2.23, *p *< 0.01). Parent's age, race and ethnicity, nativity, level of completed education, poverty level, marital or cohabitation status, and changes on mother's or father's BMI from children's 12–24 months of age were not significantly associated with changes in children's mean BMI-P (*p *> 0.05). Sensitivity tests confirmed these findings.

### Parental factors associated with change in children's BMI-P from 12 to 24 months

Table 3 shows the tests of the association of maternal factors exclusively and maternal and paternal factors combined with changes in their children's BMI-P from 12 to 24 months stratified by race and ethnicity. Within race tests showed that among White children, mean BMI-P did not significantly change from 12 to 24 months (*p* > 0.05). Tests of maternal predictors exclusively revealed that White children whose mothers completed a 4-year college degree or higher had a significant decrease in BMI-P (*β *= −46.43, 95% CI, −90.08 to −2.77, *p* = 0.04). Furthermore, when compared to White children born to parents with less than high school education, White children whose parents graduated high school or completed a 4-year college degree or higher had a significant decrease in BMI-P (*β *= −60.39, 95% CI, −115.05 to −5.72, *p *= 0.03 for high school graduates; *β *= −61.49, 95% CI, −122.44 to −0.53, *p* < 0.05 for college graduates). This association was confirmed in the tests that included the highest level of education achieved by both parents, but not in the sensitivity analyses that excluded children with imputed 12-month BMI-P measurements presented in Supplementary Table S3A. White mothers' or fathers' poverty group, cohabitation status, and BMI were not significantly associated with changes in their children's BMI-P from 12 to 24 months (*p* > 0.05).

**Table 3:** Tests of the association of maternal factors exclusively and maternal and paternal factors combined with changes in their children's BMI-P from 12 to 24 months stratified by race and ethnicity.

	Maternal factors			Parental factors^a^		
	Estimate	95% CI	*p* *	Estimate	95% CI	*p* *
A. parental predictors of change in BMI-P among non-Hispanic White children						
Child factors	*N* = 89			*N* = 80		
Child BMI-P from 12 to 24 months	−2.75	−7.16 to 1.67	0.219	−2.86	−7.26 to 1.54	0.199
Child sex						
Male (ref)	—	—	—	—	—	—
Female	−7.59	−18.05 to 2.87	0.150	−6.38	−17.01 to 4.25	0.232
Birthweight	2.44	−1.06 to 5.95	0.167	1.89	−1.70 to 5.48	0.293
Parental factors						
Nativity						
US born (ref)	—	—	—	—	—	—
Foreign born	−8.50	−27.35 to 10.35	0.367	9.10	−14.85 to 33.04	0.447
Other				−24.29	−56.82 to 8.25	0.139
Education (highest of 2)						
Less than HS	—	—	—	—	—	—
HS or equivalent	−27.77	−65.30 to 9.76	0.142	−60.39	−115.05 to −5.72	0.031
Some college but not 4-year degree	−37.67	−81.19 to 5.85	0.088	−53.56	−113.47 to 6.34	0.078
4-year degree or higher	−46.43	−90.08 to −2.77	0.038	−61.49	−122.44 to −0.53	0.048
Poverty group (highest of 2)						
≤100% FPL (ref)	—	—	—	—	—	—
>100%–200% FPL	10.10	−15.98 to 36.18	0.438	24.40	−16.09 to 64.89	0.230
>200% FPL	23.81	−4.41 to 52.02	0.096	26.86	−15.24 to 69.01	0.204
Cohabitation status						
Never married or living together (ref.)	—		—	—	—	—
Married or living together	17.27	−9.37 to 43.90	0.197	−14.25	−47.62 to 19.13	0.393
BMI time-varying						
Mother's BMI from 12 to 24 months	0.56	−0.26 to 1.38	0.173	0.48	−0.38 to 1.33	0.271
Father's BMI from 12 to 24 months	—	—	—	−0.50	−1.65, 0.63	0.373
Age at enrollment						
Mother	−0.33	−1.46 to 0.80	0.561	0.57	−1.74 to 2.88	0.621
Father	—	—	—	−0.80	−2.51 to 0.91	0.348
B. Parental predictors of change in BMI-P among Hispanic/Latinx children						
Child factors	(*N* = 123)			(*N* = 104)		
Child BMI-P from 12 to 24 months	−6.93	−10.68 to −3.19	<0.001	−7.12	−11.59 to −2.64	<0.002
Child sex						
Male (ref)	—	—	—	—	—	—
Female	6.05	−3.29 to 15.39	0.198	11.21	1.61 to 20.82	0.024
Birthweight	1.16	−1.81 to 4.13	0.433	0.48	−2.75 to 3.70	0.765
Parental factors						
Nativity						
US born (ref)	—	—	—	—	—	—
Foreign born	−3.86	−14.68 to 6.96	0.475	0.77	−14.24 to 15.78	0.918
Other	—	—	—	−7.63	−25.17 to 9.91	0.381
Education (highest of 2)						
Less than HS (ref)	—	—	—	—	—	—
HS or equivalent	2.75	−7.71 to 13.21	0.598	4.32	−7.60 to 16.24	0.465
Some college but not 4-year degree	−2.52	−18.78 to 13.75	0.756	−4.70	−21.56 to 12.16	0.573
4-year degree or higher	4.49	−21.35 to 30.33	0.727	−4.92	−27.67 to 17.83	0.662
Poverty group (highest of 2)						
≤100% FPL (ref)	—	—	—	—	—	—
>100%–200% FPL	−2.93	−13.18 to 7.32	0.565	−3.37	−15.73 to 8.99	0.582
>200% FPL	−0.11	−17.53 to 17.30	0.989	−8.63	−21.56 to 4.29	0.183
Cohabitation status						
Never married or living together (ref.)	—	—	—	—	—	—
Married or living together	1.05	−15.00 to 17.09	0.896	−4.44	−22.80 to 13.91	0.625
BMI time-varying						
Mother's BMI from 12 to 24 months	0.33	−0.42 to 1.08	0.381	0.58	−0.26 to 1.43	0.168
Father's BMI from 12 to 24 months	—	—	—	0.05	−0.94 to 1.04	0.923
Age at enrollment						
Mother	1.11	0.07 to 2.15	0.037	1.83	0.29 to 3.36	0.021
Father	—	—	—	−1.19	−2.30 to −0.08	0.036

ref., reference; HS, high school.

^a^ Parental factors included demographic characteristics of mother and father.

* *p*-value of the longitudinal mixed-effects models with maximum likelihood estimating method and unstructured covariance.

### Parental factors associated with change in Hispanic/Latinx children's BMI-P from 12 to 24 months

Table 3 provides the coefficients of the tests of maternal and maternal and paternal predictors and confirms that among Hispanic/Latinx children, BMI-P significantly decreased from 12 to 24 months. Tests of maternal predictors exclusively showed that mother's older age is associated with increases in their children's BMI-P from 12 to 24 months (*β *= 1.11, 95% CI, 0.07–2.15, *p* = 0.04). This association was consistent in the test that included maternal and paternal predictors and in the sensitivity analyses presented in Supplementary Table S3B. Tests with determinants from both parents showed that mother's older age was significantly associated with gain in children's BMI-P from 12 to 24 months (*β *= 1.83, 95% CI, 0.29–3.36, *p *= 0.02). Meanwhile, father's older age was associated with decreases in children's BMI-P from 12 to 24 months (*β *= −1.19, 95% CI, −2.30 to −0.08, *p *= 0.04). Tests including data from both parents showed that when compared with Hispanic/Latino males, mean BMI-P of Hispanic/Latina females significantly increased from 12 to 24 months (*β *= 11.21, 95% CI, 1.61–20.82, *p* = 0.02). Parent's nativity, level of completed education, poverty level, marital or cohabitation status, and changes in parent's BMI were not significantly associated with changes in Hispanic/Latinx children's BMI-P from 12 to 24 months (*p* > 0.05). Sensitivity analyses confirmed these findings.

## Discussion

The first 2 years of life are known to be a critical period with important health consequences for future health; yet, information of children's growth in very early ages, particularly of racial and ethnic diverse children, remains limited. Childhood obesity rates have not plateaued in the United States. Data from 2015 to 2016 showed a sharp increase in obesity prevalence among children aged 2–5 years (25). These alarming increases highlight the need to improve the identification of children at risk for excessive weight gain during the first 2 years of life, when prevention may be more effective. To this end, this study advances our understanding of growth in early childhood using current data from a diverse sample of children and examining determinants from mothers and both parents. Findings revealed that, when assessing all children's growth from 12 to 24 months of age, BMI-P decreased. However, changes in BMI-P suggest that racial/ethnic disparities between Hispanic/Latinx and White children may begin in the first year of life. Analyses including only maternal demographic factors showed that having a Hispanic/Latinx mother and higher birthweight was associated with gain in BMI-P from 12 to 24 months. Racial and ethnicity-stratified analyses revealed that among White children, higher parental education, particularly having at least one parent graduate from high school or from a 4-year college or higher, was associated with greater BMI-P decreases during the first year of life in relationship with less than high school education. Notably, higher parental education achievement did not afford the same benefits to Hispanic/Latinx children. It was also noted that the inclusion of paternal predictors in the models helped identify factors that were not associated with children's BMI-P changes in the model that examined the contribution of maternal factors. Together, these findings highlight the importance of increasing representation of racial and ethnic diverse families in research and the urgent need to also consider the contributions of paternal determinants to child health outcomes, not instead of but in addition to the mothers.

A recent study showed that in the United States, trajectories and precursors of excessive weight gain from birth to 9 years of age vary by race and ethnicity (7). Our findings confirm that racial and ethnic differences in children's growth, and the factors that influence their growth, begin early in life. A better understanding of the racial and ethnicity-specific determinants associated with weight changes is urgently needed to inform culturally grounded obesity prevention strategies.

In the analyses of comparisons within White children, the level of education completed by their mother and the highest level of education completed by either parent, specifically having graduated from high school or college, was associated with decreases in children’s BMI-P from 12 to 24 months. A systematic review that examined data from the 27 richest countries suggested that parental education is a stronger indicator of child and adolescent weight status than income and/or occupation (26). This review suggested that the association can be attributed to healthier lifestyles in homes where parents have higher education levels (26). However, the association of parental educational achievement with children's optimal growth is not consistent. Previous studies conducted with older children have shown that having a parent with higher education attainment was associated with lower obesity risks among White, but not among Black, youth (27, 28). This is consistent with the diminishing return hypothesis that poses that the effects of socioeconomic achievement are not uniform across diverse racial and ethnic individuals (10). Our findings expand this literature by evidencing that racial and ethnic differences in the association of parental education attainment with children's optimal growth were present from very early ages and influenced White, but not Hispanic/Latinx, children. We hypothesize that persistent racial and ethnic inequalities and discrimination experiences may explain the lack of health benefits observed among children born to Hispanic/Latinx parents. For example, it has been documented that racial and ethnic minority parents with higher educational achievements, particularly first-generation college graduates, earn less than their White counterparts (29). As a result of the payment gap, it can be presumed that the offspring of Hispanic/Latinx parents who achieved higher levels of education received only a fraction of the benefits than higher education introduced to their White counterparts.

Comparisons within the subsample of Hispanic/Latinx children showed that mothers older age was associated with higher BMI-P in children, while father's older age was associated with lower BMI-P. A previous study showed that maternal age below 25 or above 35 years was associated with higher obesity risks for their offspring (30). However, the association of maternal age with offspring weight gain is not always consistent. In a sample of White children living in New Zealand, researchers found that children born to mothers over 35 years old were about 0.5 cm taller and had 13% lower adiposity than the offspring of mothers aged less than 30 years (31). We were not able to find previous studies that included Hispanic/Latino fathers and examined the impact of fathers’ age on children's obesity risks. This is likely attributed to the lower representation of fathers in research and lack of examinations of father's age as a covariate. We hypothesized that the association of parental age with changes in children's BMI-P after the first year of life is likely due to differences in parental feeding styles and socioeconomic resources. Older Hispanic/Latinx mothers may be more likely to sooth a fussy baby with food than their younger counterparts. In the case of older Hispanic/Latinx fathers, we hypothesize that they may be more financially stable than their younger counterparts. A previous study conducted with nationally representative data found that a lower socioeconomic status in early childhood predicted obesity at age 4, particularly among children with Hispanic/Latinx mothers (32). There is a lack of knowledge about the fathers’ determinants that contribute to childhood obesity risks, particularly among racial/ethnic minority families. As Hispanic/Latinx women and men continue to postpone fertility, it is important to better understand the potential contribution to child obesity risks that may be attributed to mothers' and fathers' age at the time of birth.

Models of Hispanic/Latinx children that included information from both parents suggested that when compared with males, females were more likely to experience increases in BMI-P from 12 to 24 months, a period of life when BMI-P is expected to decrease (33). Previous studies have shown that sex differences in older ages are contrary to our findings. Among White and Hispanic/Latinx children aged 2–5 years old, prevalence of obesity is higher among males than females (34). Further research is needed to further examine sex differences in BMI-P gains among Hispanic/Latinx children in early childhood.

Contrary to our hypotheses, parent's change in BMI-P was not associated with children's change in BMI-P from 12 to 24 months. Several studies have found that childhood BMI-P gains and obesity risks are greatly influenced by the weight status of mothers and fathers (35). However, important variations in the association suggest variations by children's age, family socioeconomic status, parental weight status, and, in the United States, race and ethnicity (35–37).

In the United States, the intergenerational transmission of obesity appears to be greater when examining maternal influences and present most often among White children than racial and ethnic minorities, particularly in families with older parents and higher socioeconomic status (36–38). We speculate the demographics of our sample and younger age of children at the time of the assessment may explain the lack of an association. These null findings support previous studies that suggested that intergenerational transmission of obesity is mostly attributable to behavioral, social, and environmental factors rather than biological differences, and are consequentially preventable.

Studies that use more precise indicators of adiposity have recognized that the ability of the current BMI-P cut-off recommendations to identify youth with excess body fatness and cardiometabolic risks varies by race/ethnicity (39). In nationally representative samples of US youth, studies have shown that non-Hispanic Black youth have significantly lower percentage of body fat for BMI than non-Hispanic White youth; meanwhile, Asian and Mexican American or Hispanic youth have higher fatness (39, 40). Higher fatness at similar levels of BMI is associated with higher cardiometabolic risks among Asian and Mexican American adults (41). When compared with other ethnic/racial groups in the United States, both Mexican American and Asian adults had a higher percent of body fat and metabolic syndrome at similar BMI levels (41). In response to the evidence of increased cardiometabolic risks at lower BMI cut-off points documented among Asian adults, the World Health Organization introduced population-specific BMI cut-off criteria for overweight and obesity (42). However, to date, population-specific recommendations have not been issued for Asian children or adolescents and Hispanic/Latinx children or adults. Race- and ethnicity-specific cut-off values (43) along with unique sociocultural measurements (44, 45) are needed to improve researchers’ and clinicians' ability to prevent, monitor, and treat obesity and obesity-related comorbidities among racial and ethnic minority children.

To our knowledge, this is among the first studies to include data collected with a racial and ethnic diverse sample of both mothers and fathers in the assessments of changes in BMI-P after the first year of life, a critical age for future obesity risks. A recent systematic review examining the inclusion of fathers in childhood obesity research found that fathers were included only in less than 10% of studies (18). In our study, the inclusion of maternal predictors and combined maternal and paternal determinants elucidated important differences in risk factors affecting children's BMI-P changes in early life. Overall models showed that considering father's educational attainment, poverty level, and age, along with mother's demographics attenuated the association of children's birthweight and mothers’ Hispanic/Latinx background with children's BMI-P change from 12 to 24 months of age. These associations were also attenuated in our sensitivity analyses presented in the Supplementary Tables, further suggesting lower robustness.

Findings of this study should be interpreted with caution in the context of the sample limitations. The study included a racial and ethnic diverse sample of predominantly low-income families. Thus, findings are not nationally representative and should be interpreted in the context of the large proportion White families with high education and Hispanic/Latinx with low education attainment who participated in the study. The limited number of African American/Black families who participated in the 24-month examination is also a key limitation that precluded the inclusion of this important racial minority in our stratified analyses. The early-life parental predictors of changes in BMI-P identified are not necessarily cause of obesity. Rather, factors are associated with children's probability of increases and decreases in BMI-P from 12 to 24 months. Although we examined multiple maternal and paternal predictors, lurking and unexamined variables (e.g., breastfeeding status, social determinants of health, timing of introduction to solids, parenting and feeding style, etc.) may explain the observed differences by race and ethnicity and the associations identified.

In conclusion, our results highlight the importance of increasing representation of racial and ethnic minorities in research and considering the influence of social determinants influencing the lives of both mothers and fathers in analyses of BMI-P changes during early life. Our findings suggest that higher levels of parental education were associated with greater reductions in BMI-P among White, but not Hispanic/Latinx, children. Among Hispanic/Latinx, girls and children born to older mothers and/or younger fathers were more likely to experience increases in BMI-P from 12 to 24 months than boys and children born to younger mothers and older fathers. Further research is needed to better understand the race- and ethnicity-specific mechanisms through which maternal and paternal social determinants influence disparities in children's growth and obesity risks in early life.

## Data Availability

The datasets for this article are not publicly available due to concerns regarding participant/patient anonymity. Requests to access the datasets should be directed to The Eunice Kennedy Shriver National Institute of Child Health and Human Development Data and Specimens Hub (NICHD/DASH).
