# Supplementary material for: Parental determinants associated with early growth after the first year of life by race and ethnicity
Source: Front Pediatr. 2023 Jul 25;11:1213534. doi: 10.3389/fped.2023.1213534 (PMC10411553; doi:10.3389/fped.2023.1213534)
Supplement: Supplementary file 1 [file Table1.pdf]

Supplementary Table 1. Child, Mother and Father Demographic Characteristics, Overall and by Race and Ethnicity

	Overall	Non-Hispanic White	Non-Hispanic Black	Hispanic/ Latinx	Other	*p**
**CHILD CHARACTERISTICS**	n (%) / mean ± *sd*					
**Child Sex**	231	73	23	101	34	
Male	117 (50.65)	35 (47.95%)	13 (56.52%)	52 (51.49%)	17 (50.00%)	0.904
Female	114 (49.35)	38 (52.05%)	10 (43.48%)	49 (48.51%)	17 (50.00%)	
**Child Birthweight**						
Low	29 (12.55)	9 (12.33%)	4 (17.39%)	11 (10.89%)	5 (14.71%)	
Normal	176 (76.19)	54 (73.97%)	16 (69.57%)	78 (77.23%)	28 (82.35%)	0.5715
Macrosomia	26 (11.26)	10 (13.70%)	3 (13.04%)	12 (11.88%)	1 (2.94%)	
**Child weight status**						
Child BMI-P at 12 months	61.61 (30.12)	61.75 ± 28.47	49.73 ± 35.80	62.42 ± 31.63	65.85 ± 24.84	0.384
Overweight or obesity at 12 months (BMI-P >85th)	58 (25.11)	19 (26.03%)	3 (13.04%)	30 (29.70%)	6 (17.65%)	0.364
Child BMI-P at 24 months	51.79 (32.68)	51.20 ± 34.69	37.70 ± 29.61	55.11 ± 31.86	51.27 ± 31.22	0.584
Overweight or obesity at 24 months (BMI-P >85th)	49 (21.21)	17 (23.29%)	3 (13.04%)	23 (22.77%)	6 (17.65%)	0.921
**PARENTAL CHARACTERISTICS**						
Mother's age	(27.20 ± 5.66)	30.75 ± 4.73	25.91 ± 5.75	25.08 ± 4.71	26.28 ± 6.38	<.001
Father's age	(29.77 ± 7.03)	32.47 ± 6.38	28.60 ± 6.33	28.10 ± 6.79	29.74 ± 7.99	<.001
**Mother's Education**						
Less than High School (HS)	50 (21.65)	2 (2.74%)	4 (17.39%)	40 (39.60%)	4 (11.76%)	
HS or equivalent	79 (34.20)	8 (10.96%)	9 (39.13%)	45 (44.55%)	17 (50.00%)	
Some college but not 4-yr degree	43 (18.61)	17 (23.29%)	7 (30.43%)	11 (10.89%)	8 (23.53%)	<.001
4-yr degree or higher	53 (22.94)	43 (58.90%)	3 (13.04%)	3 (2.97%)	4 (11.76%)	
Other	6 (2.60)	3 (4.11%)	0 (0.00%)	2 (1.98%)	1 (2.94%)	
**Father's Education**						
Less than High School (HS)	58 (25.11)	5 (6.85%)	5 (21.74%)	45 (44.55%)	3 (8.82%)	
HS or equivalent	89 (38.53)	17 (23.29%)	10 (43.48%)	46 (45.54%)	16 (47.06%)	<.001
Some college but not 4-yr degree	28 (12.12)	6 (8.22%)	6 (26.09%)	7 (6.93%)	9 (26.47%)	
4-yr degree or higher	56 (24.24)	45 (61.64%)	2 (8.70%)	3 (2.97%)	6 (17.65%)	
**Mother's Poverty Group**						
<=100% FPL	64 (27.71)	3 (4.11%)	11 (47.83%)	37 (36.63%)	13 (38.24%)	
>100 to 200% FPL	77 (33.33)	12 (16.44%)	5 (21.74%)	50 (49.50%)	10 (29.41%)	<.001
>200% FPL	90 (38.96)	58 (79.45%)	7 (30.43%)	14 (13.86%)	11 (32.35%)	
**Father's Poverty Group**						
<=100% FPL	62 (26.84)	3 (4.11%)	7 (30.43%)	42 (41.58%)	10 (29.41%)	
>100 to 200% FPL	56 (24.24)	13 (17.81%)	4 (17.39%)	33 (32.67%)	6 (17.65%)	<.001
>200% FPL	113 (48.92)	57 (78.08%)	12 (52.17%)	26 (25.74%)	18 (52.94%)	
**Marital or Cohabitation status**						
Not married or living together	27 (11.69)	3 (4.11%)	10 (43.48%)	9 (8.91%)	5 (14.71%)	<.001
Married or Living together	204 (88.31)	70 (95.89%)	13 (56.52%)	92 (91.09%)	29 (85.29%)	

Supplementary Table 1 (continuation)

	Overall	Non-Hispanic White	Non-Hispanic Black	Hispanic/ Latinx	Other	p*
**PARENTAL CHARACTERISTICS**	n (%) / mean ± *sd*					
**Mother's Race and Ethnicity**						
Non-Hispanic White	81 (35.06)	73 (100.00%)	0 (0.00%)	0 (0.00%)	8 (23.53%)	<.001
Non-Hispanic Black	26 (11.26)	0 (0.00%)	23 (100.00%)	0 (0.00%)	3 (8.81%)	
Hispanic/ Latinx	118 (51.08)	0 (0.00%)	0 (0.00%)	101 (100.00%)	17 (50.00%)	
Other	6 (2.60)	0 (0.00%)	0 (0.00%)	0 (0.00%)	6 (17.65%)	
**Father's Race and Ethnicity**						
Non-Hispanic White	84 (36.36)	73 (100.00%)	0 (0.00%)	0 (0.00%)	11 (32.35%)	<.001
Non-Hispanic Black	29 (12.55)	0 (0.00%)	23 (100.00%)	0 (0.00%)	6 (17.65%)	
Hispanic/ Latinx	106 (45.89)	0 (0.00%)	0 (0.00%)	101 (100.00%)	5 (14.71%)	
Other	12 (5.19)	0 (0.00%)	0 (0.00%)	0 (0.00%)	12 (35.29%)	
**Mother's Nativity**						
US born	140 (60.61)	68 (93.15%)	22 (95.65%)	22 (21.78%)	28 (82.35%)	<.001
Foreign born	91 (39.39)	5 (6.85%)	1 (4.35%)	79 (78.22%)	6 (17.65%)	
**Father's Nativity**						
US born	144 (62.34)	70 (95.89%)	21 (91.30%)	23 (22.77%)	30 (88.24%)	<.001
Foreign born	87 (37.66)	3 (4.11%)	2 (8.70%)	78 (77.23%)	4 (11.76%)	
**Mother's Weight Status**						
BMI (child's age 12 months)	28.28 (6.21)	27.86 ± 6.63	30.74 ± 8.31	28.20 ± 5.08	28.08 ± 7.10	0.416
Overweight or Obesity at 12 months (BMI>25 kg/m2)	141 (61.04)	43 (58.90%)	18 (78.26%)	62 (61.39%)	18 (52.94%)	0.360
BMI (child's age 24 months)	28.23 (5.93)	27.95 ± 5.86	25.96 ± 5.51	29.04 ± 6.09	27.92 ± 6.05	0.546
Overweight or Obesity at 24 months (BMI>25 kg/m2)	136 (58.87)	41 (56.16%)	14 (60.87%)	57 (56.44%)	22 (64.71%)	0.339
**Father's Weight Status**						
BMI (child's age 12 months)	28.76 (4.79)	29.03 ± 4.89	29.76 ± 5.99	28.46 ± 4.48	28.45 ± 4.88	0.718
Overweight or Obesity at 12 months (BMI>25 kg/m2)	158 (68.40)	52 (71.23%)	16 (69.57%)	70 (69.31%)	20 (58.82%)	-
BMI (child's age 24 months)	28.67 (5.12)	28.87 ± 4.88	27.80 ± 6.17	28.79 ± 5.02	28.24 ± 5.91	0.932
Overweight or Obesity at 24 months (BMI>25 kg/m2)	139 (60.17)	47 (64.38%)	17 (73.91%)	55 (54.46%)	19 (55.88%)	0.256

**p-value* of comparisons across racial and ethnic groups carried out using chi-square/ Fisher's exact tests (for categorical variables), and ANOVA (for continuous variables).

sd = standard deviation; BMI-P = Age and sex -adjusted Body Mass Index Percentile; HS= High School; FPL= Federal Poverty Line.

Supplementary Table 2. Association of changes in children's age and sex -adjusted body mass index percentile (BMI-P) from 12 to 24 months with maternal factors and parental factors

	**MATERNAL FACTORS**			**PARENTAL FACTORS***		
	Estimate	95% CI	$P^{\pm}$	Estimate	95% CI	$P^{\pm}$
**CHILD FACTORS**		**(N=231)**			**(N=231)**	
Child BMI-P from 12 to 24 months	-5.87	(-8.59, -3.14)	<0.001	-5.57	(-8.28, -2.87)	<0.001
**Child Sex**						
Male (ref)	-	-	-	-	-	-
Female	1.90	(-4.86, 8.66)	0.578	2.95	(-3.72, 9.61)	0.383
**Birthweight**	1.97	(-0.14, 4.08)	0.067	1.87	(-0.28, 4.01)	0.088
**PARENTAL FACTORS**						
**Race**						
Non-Hispanic White (ref)	-		-	-	-	-
Non-Hispanic Black	-8.83	(-21.81, 4.14)	0.180	-6.21	(-20.07, 7.65)	0.376
Hispanic/ Latinx	10.67	(-0.89, 22.22)	0.070	3.88	(-9.98, 17.75)	0.580
Other	-15.32	(-36.99, 6.36)	0.164	5.10	(-6.77, 16.97)	0.396
**Nativity**						
US born (ref)	-		-	-	-	-
Foreign born	-5.43	(-15.05, 4.19)	0.265	4.80	(-5.61, 15.21)	0.521
Other	-		-	-1.14	(-13.87, 11.59)	0.859
**Education (highest of 2)**						
Less than High School (HS)	-		-	-	-	-
HS or equivalent	3.31	(-6.34, 12.97)	0.497	1.72	(-9.24, 12.68)	0.756
Some college but not 4-yr degree	0.33	(-12.63, 13.28)	0.960	1.04	(-12.56, 14.64)	0.879
4-yr degree or higher	-3.49	(-19.23, 12.26)	0.661	1.29	(-14.05, 16.63)	0.867
**Poverty Group (highest of 2)**						
<=100% FPL (ref)	-		-	-	-	-
>100 to 200% FPL	-2.67	(-11.71, 6.36)	0.558	-1.94	(-12.28, 8.40)	0.710
>200% FPL	0.30	(-12.14, 12.75)	0.962	-3.80	(-14.72, 7.11)	0.491
**Cohabitation status**						
Never married or living together (ref.)	-		-	-		-
Married or living together	8.64	(-3.37, 20.66)	0.157	8.17	(-3.83, 20.17)	0.179
**BMI time-varying**						
Mother's BMI from 12-24 months	0.29	(-0.22, 0.79)	0.267	0.34	(-0.18, 0.85)	0.198
Father's BMI from 12-24 months				-0.09	(-0.75, 0.57)	0.787
**Age at enrollment**						
Mother	0.27	(-0.49, 1.02)	0.485	0.63	(-0.45, 1.71)	0.253
Father				-0.59	(-1.41, 0.23)	0.156

*Parental factors included demographic characteristics of mother and father

$^{\pm}$*P-value* of the longitudinal mixed-effects models with maximum likelihood estimating method and unstructured covariance

CI = Confidence Interval; ref. = reference; BMI-P = Age and sex -adjusted Body Mass Index Percentile; HS= High School; FPL= Federal Poverty Line.

Supplementary Table 3. Tests of the association of maternal factors exclusively and maternal and paternal factors combined with changes in their children's BMI-P from 12 to 24 months stratified by race and ethnicity.

TABLE S3A. Parental Predictors of Change in BMI-P among non-Hispanic White Children						
	MATERNAL FACTORS			PARENTAL FACTORS*		
	Estimate	95% CI	P±	Estimate	95% CI	P±
**CHILD FACTORS**		**(N=77)**			**(N=73)**	
Child BMI-P from 12 to 24 months	-4.07	(-8.70, 0.57)	0.084	-3.49	(-8.13, 1.15)	0.137
**Child Sex**						
Male (ref)	-	-	-	-	-	-
Female	-8.12	(-18.73, 2.48)	0.129	-5.97	(-16.59, 4.64)	0.262
Birthweight	2.08	(-1.32, 5.48)	0.223	1.60	(-1.85, 5.05)	0.353
**PARENTAL FACTORS**						
**Nativity**						
US born (ref)	-	-	-	-	-	-
Foreign born	-14.99	(-34.55, 4.58)	0.129	5.77	(-20.43, 31.97)	0.658
Other				-26.39	(-57.57, 4.78)	0.095
**Education (highest of 2)**						
Less than High School (HS)	-	-	-	-	-	-
HS or equivalent	-16.44	(-54.91, 22.03)	0.393	-37.64	(-101.23, 25.95)	0.238
Some college but not 4-yr degree	-28.86	(-73.05, 15.33)	0.194	-34.24	(-102.50, 34.02)	0.316
4-yr degree or higher	-36.88	(-81.60, 7.84)	0.103	-39.69	(-109.54, 30.16)	0.257
**Poverty Group (highest of 2)**						
<=100% FPL (ref)	-	-	-	-	-	-
>100 to 200% FPL	4.54	(-25.17, 34.24)	0.759	11.45	(-42.66, 65.55)	0.671
>200% FPL	13.97	(-17.26, 45.21)	0.371	8.85	(-46.25, 63.95)	0.747
**Cohabitation status**						
Never married or living together (ref.)	-		-	-	-	-
Married or living together	23.19	(-2.88, 49.27)	0.080	0.59	(-39.69, 40.87)	0.977
**BMI time-varying**						
Mother's BMI from 12-24 months	0.37	(-0.46, 1.21)	0.367	0.48	(-0.35, 1.32)	0.250
Father's BMI from 12-24 months	-	-	-	-0.85	(-1.98, 0.27)	0.132
**Age at enrollment**						
Mother	-0.39	(-1.52, 0.75)	0.498	0.57	(-1.64, 2.78)	0.603
Father	-	-	-	-0.98	(-2.63, 0.66)	0.234

*Parental factors included demographic characteristics of mother and father

±*P-value* of the longitudinal mixed-effects models with maximum likelihood estimating method and unstructured covariance

CI = Confidence Interval; ref. = reference; BMI-P = Age and sex -adjusted Body Mass Index Percentile; HS= High School; FPL= Federal Poverty Line.

Supplementary Table 3 (continuation)

Table S3B. Parental Predictors of Change in BMI-P among Hispanic/Latinx Children

	MATERNAL FACTORS			PARENTAL FACTORS*		
	Estimate	95% CI	P±	Estimate	95% CI	P±
**CHILD FACTORS**		**(N=116)**			**(N=101)**	
Child BMI-P from 12 to 24 months	-7.72	(-11.55, -3.89)	<0.001	-7.90	(-12.43, -3.37)	<0.001
**Child Sex**						
Male (ref)	-	-	-	-	-	-
Female	6.45	(-3.23, 16.12)	0.186	11.89	(2.03, 21.75)	0.020
Birthweight	1.15	(-1.89, 4.19)	0.449	0.35	(-2.92, 3.61)	0.830
**PARENTAL FACTORS**						
**Nativity**						
US born (ref)	-	-	-	-	-	-
Foreign born	-3.81	(-14.98, 7.35)	0.494	0.30	(-15.01, 15.60)	0.969
Other	-	-	-	-9.28	(-27.29, 8.72)	0.301
**Education (highest of 2)**						
Less than High School (HS)	-	-	-	-	-	-
HS or equivalent	3.26	(-7.66, 14.19)	0.549	5.73	(-6.71, 18.18)	0.355
Some college but not 4-yr degree	-2.67	(-19.51, 14.17)	0.750	-3.25	(-20.51, 14.02)	0.704
4-yr degree or higher	5.58	(-20.79, 31.95)	0.671	-3.17	(-26.41, 20.06)	0.783
**Poverty Group (highest of 2)**						
<=100% FPL (ref)	-	-	-	-	-	-
>100 to 200% FPL	-2.97	(-13.55, 7.61)	0.574	-3.55	(-16.05, 8.95)	0.567
>200% FPL	-0.74	(-18.64, 17.17)	0.934	-10.12	(-23.66, 3.42)	0.138
**Cohabitation status**						
Never married or living together (ref.)	-	-	-	-	-	-
Married or living together	0.66	(-15.68, 17.00)	0.935	-5.22	(-23.79, 13.34)	0.570
**BMI time-varying**						
Mother's BMI from 12-24 months	0.38	(-0.40, 1.15)	0.332	0.62	(-0.26, 1.49)	0.161
Father's BMI from 12-24 months	-	-	-	-0.12	(-1.17, 0.93)	0.817
**Age at enrollment**						
Mother	1.15	(0.09, 2.22)	0.035	1.89	(0.33, 3.47)	0.020
Father	-	-	-	-1.16	(-2.29, -0.04)	0.043

*Parental factors included demographic characteristics of mother and father

±*P-value* of the longitudinal mixed-effects models with maximum likelihood estimating method and unstructured covariance

CI = Confidence Interval; ref. = reference; BMI-P = Age and sex -adjusted Body Mass Index Percentile; HS= High School; FPL= Federal Poverty Line.
